# Target guided isolation, *in-vitro* antidiabetic, antioxidant activity and molecular docking studies of some flavonoids from *Albizzia Lebbeck Benth*. bark

**DOI:** 10.1186/1472-6882-14-155

**Published:** 2014-05-13

**Authors:** Danish Ahmed, Vikas Kumar, Manju Sharma, Amita Verma

**Affiliations:** 1 Department of Pharmaceutical Sciences, Faculty of Health Sciences, Sam Higginbottom Institute of Agriculture, Technology & Sciences, Deemed University, Allahabad, Uttar Pradesh, India; 2 Department of Pharmacology, Faculty of Pharmacy, Jamia Hamdard, New Delhi, India; 3 Department of Pharmacology, Hamdard Institute of Medical Sciences & Research, Jamia Hamdard, New Delhi, India

## Abstract

**Background:**

*Albizzia Lebbeck Benth.* is traditionally important plant and is reported to possess a variety of pharmacological actions. The present research exertion was undertaken to isolate and characterized the flavonoids from the extract of stem bark of *Albizzia Lebbeck Benth.* and to evaluate the efficacy of the isolated flavonoids on in-vitro models of type-II diabetes. Furthermore, the results of *in-vitro* experimentation inveterate by the molecular docking studies of the isolated flavonoids on α-glucosidase and α-amylase enzymes.

**Methods:**

Isolation of the flavonoids from the methanolic extract of stem bark of *A. Lebbeck Benth* was executed by the Silica gel (Si) column chromatography to yield different fractions. These fractions were then subjected to purification to obtain three important flavonoids. The isolated flavonoids were then structurally elucidated with the assist of ^1^H-NMR, ^13^C-NMR, and Mass spectroscopy. In-vitro experimentation was performed with evaluation of α-glucosidase, α-amylase and DPPH inhibition capacity. Molecular docking study was performed with GLIDE docking software.

**Results:**

Three flavonoids, (1) 5-deoxyflavone (geraldone), (2) luteolin and (3) Isookanin were isolated from the EtOAc fraction of the methanolic extract of *Albizzia lebbeck Benth* bark. (ALD). All the compounds revealed to inhibit the α-glucosidase and α-amylase enzymes in *in-vitro* investigation correlating to reduce the plasma glucose level. Molecular docking study radically corroborates the binding affinity and inhibition of α-glucosidase and α-amylase enzymes.

**Conclusion:**

The present research exertion demonstrates the anti-diabetic and antioxidant activity of the important isolated flavonoids with inhibition of α-glucosidase, α-amylase and DPPH which is further supported by molecular docking analysis.

## Background

Type 2 diabetes mellitus (T2D) is one of the fastest growing epidemics of our time [1-3]. This disease affected nearly 150 million adults worldwide in 2000. T2D is characterized by decreased insulin sensitivity leading to insulin resistance in its target tissues (mainly liver, skeletal muscle and adipose tissues) [4-6]. On the other hand, impaired glucose-induced insulin secretion (GIIS) with a decrease in pancreatic -cell mass will eventually lead to chronic hyperglycaemia [7]. Both genetic and environmental factors are involved in the aetiology of T2D and dysfunction of fatty-acid (FA) metabolism appears to be an early key event leading to insulin resistance [6].

Insulin dependent diabetes mellitus (IDDM) or juvenile-onset diabetes accounts for about 10% of diabetes. The main symptoms include high blood sugar, excessive thirst, frequent urination, increased appetite, fatigue, weight loss, poor wound healing, blurred vision etc. the only treatment of this type available is insulin injections. The risk of hypoglycemia is greatly increased. In 2011 about 366 million people suffered with diabetes globally and this is expected to increase to 552 million by 2030 [8]. One recent study by ICMR-INDIA reported that about 62.4 million type-II diabetic people are from India. This statistics are expected to increase to 101 million by the year 2030 [9].

A change in diet, lifestyle and exercise will help to a great extent in management of diabetes at the early stages with little lesser impact at the later stages of life. Quite a lot of minerals have been found to benefit diabetes either due to deficiencies or their favorable effect on metabolism of glucose [10].

Plant-based products have been popular all over the world for centuries. In diabetics, some herbal alternatives are proven to provide symptomatic relief and assist in the prevention of the secondary complications of the disease. Some herbs have also been proven to help in the regeneration of beta cells and in triumph over insulin resistance. Apart from normalizing the blood glucose level, some the Indian Medicinal plants are also reported to possess antioxidant and cholesterol lowering action [3].

Therefore, herbal medication is the most widespread used unconventional therapy for diabetes treatment. Alternatives are considered necessary because of inability of current therapies to contribute normoglycemia and prevention of diabetic complications. Due to high cost of modern treatment and medications and dependency of about 85-95% of rural population in developing countries on traditional medicines for their primary health care necessitates the alternative strategies for the prevention and treatment of diabetes. Moreover herbs are known for their safety, efficacy, cultural acceptability and lesser side effects besides maintaining normal blood glucose level in diabetic patients [3].

Preparation of standardized medicinal herbs is urgently needed for future studies and therapies. To date, over 600 traditional plant treatments for diabetes have been reported but only a small number of these have received scientific and clinical evaluation to assess their efficacy. However, the hypoglycemic effect of some herbal extracts have been confirmed in human and animal models of type-II diabetics and some of the conventional drugs have been derived from the active molecules of these medicinal plants. For example, metformin a less toxic biguanide and potent oral hypoglycemics was developed from plant *Galgea officinals*[11]. The need of basic information about the availability of antidiabetic plants in India for researchers for their scientific quest is fanatically felt.

Due to capability of antioxidants to assuage oxidative stress in cells and helps in the prevention and treatment of many diseases of humans, the exploration for antioxidants has attracted much attention in the past decades and many medicinal plants are considered to have colossal antioxidant potential [12].

*Albizzia Lebbeck Benth*. is traditionally important medicinal plant. *Albizia lebbeck Benth*. (Mimosaceae), commonly known as Sirisa in Sanskrit, is a tall, unarmed, and deciduous tree distributed throughout India from the plains up to 900 m in the Himalayas. In Siddha system of medicine the bark and flowers of this plant are used to treat arthritis [13]. The stem bark of *Albizia lebbeck Benth* (ALD). is used to treat diarrhea [14], poisoning, edema, asthma and bronchitis [15]. The leaves of this plant contain alkaloids, flavonoids, tannins and saponins which have brilliant therapeutic importance [16].

A research work indicates that flavonoids isolated from *Albizzia Julibrissin* showed significant antioxidant activity. Present research exertion explores the possible hypoglycemic and lipid lowering properties of some of the flavonoids which were previously isolated and characterized from bark of *Albizzia Julibrissin* have shown to produce powerful antioxidant effects [17].

In the present study we hypothesized that the function of the bark for the treatment of type –II diabetes may be mediated with the compounds in the plants with antioxidant activity, because oxidative stress is considered to be major cause in the development of type-II diabetes [18]. An in-vitro evaluation was therefore undertaken and antioxidant and antidiabetic activities of the isolated flavonoids were evaluated by and α-glucosidase, α-amylase and DPPH inhibition assay. Furthermore, the mechanism of action of the isolated compounds was explored by molecular docking analysis.

## Methods

### General experimental procedures

^1^H and ^13^C NMR spectra were recorded on a Bruker Advance II NMR spectrometer at 400 MHz for proton and 100 MHz for carbon utilizing TMS as internal standard. Solvent used was DMSO-d_6._ EIMS were recorded WATERS, Q-TOF micromass.

### Plant material

The bark of *Albizzia Lebbeck Benth.* was collected in the month of December 2012 from Herbal Garden of Department of Pharmaceutical Sciences, Faculty of Health Sciences, SHIATS-Deemed University, Allahabad, Uttar Pradesh. The botanical identity of the plant was confirmed with the help of Pharmacognosist, Department of Pharmaceutical Sciences, Faculty of Health Sciences, SHIATS-Deemed University, Allahabad, Uttar Pradesh. The voucher specimen was deposited in the Department of Pharmacognosy, Faculty of Health Sciences, SHIATS-Deemed University, Allahabad, Uttar Pradesh. (Voucher No. ALD/B/2012/12/0018).

### Preparation of extracts

The bark of *Albizzia Lebbeck Benth.* was chopped into small pieces, cleaned, powdered, air-dried, sieved (Mesh size = 40) and stored in air tight container at room temperature. The powdered plant material (14.2 kgs) was then extracted sequentially with Ethyl Acetate (EtOAc), dichloromethane (CH_2_Cl_2_), and methanol (MeOH) using soxhlation method. The extract (1.89 kg) was concentrated to dryness using Rotary Evaporator (Rotavapor, R-210/R-215, Buchi). The percentage yield of Ethyl Acetate (EtOAc), dichloromethane (CH_2_Cl_2_), and methanol extract was found to be 0.27%, 1.63% and 16.39% respectively.

### Phytochemical analysis

#### *Preliminary phytochemical evaluation*

In order to confirm the presence of different types of compounds, some important colorimetric tests were used to reveal the families of the compounds. The presence of saponins, flavonoids and reducing compounds were revealed by Shibata Test and Fehling’s test [19]. The Presence of tannins was confirmed using FeCl_3_ and Stiasny’s reagent according to the Soro et al. [20].

### Phytochemical screening of *Albizzia Lebbeck Benth.* Stem bark. (ALD)

As our research exertion was focused on the isolation of flavonoids, phytochemical screening of *Albizzia Lebbeck Benth*. bark was carried out for the presence of flavonoids.

### Test for flavonoids

Presence of flavonoids was inveterate by the method developed by Saeed et al. [21]. Filtrate was obtained by suspending 50 mg of the ALD extract in 100 ml of distilled water. From the filtrate 10 ml was pipette out and 5 ml of the dilute ammonia solution was added to the filtrate followed by few drops of concentrated H_2_SO_4_. Yellow color confirms the presence of flavonoids.

### Determination of total flavonoid content

Total flavonoid content was determined following a method by Perk *et al.*[22]. 0.3 ml of the extract, 3.4 ml of 30% methanol, 0.15 ml of NaNO_2_ (0.5 M) and 0.15 ml of AlCl_3_.6H_2_O (0.3 M) were mixed in a 10 ml test tube. 1 ml of NaOH (1 M) was added after an interval of 5 minutes. The solution was then mixed well and the absorbance was measured against a reagent blank at 506 nm with UV spectrophotometer type Shimadzu UV–vis 1800. The flavonoid content was calculated from a quercetin standard curve and expressed in mg quercetin equivalent per g of dry weight (DW).

### Extraction and isolation of compounds 1–3

**Figure 1:**
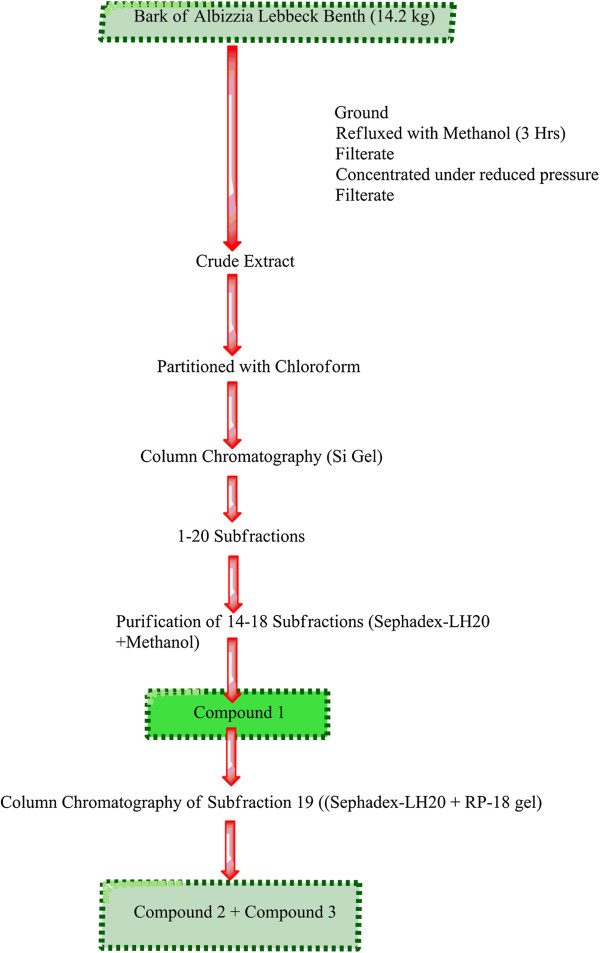
**Schematic representation for preparation of isolated compounds from stem bark of *Albizzia Lebbeck Benth*.**

The isolation of the compounds was performed according to the method developed by Jung *et al.*[17]. The stem bark (14.2 kg) of the Albizzia Lebbeck Benth. Benth. was refluxed with methanol of three hours. (14 L x3). After refluxing the bark with methanol, the total filtrate was concentrated to dryness in vacuum at 40°C leaving behind the methanolic extract (1.89 kg). The methanolic extract was then suspended in water and partitioned with Chloroform: methanol in sequence (100, 90:10, 80:20, 70:30, 60:40, 50:50, 40:60, 30:70, 20:80, 10:90, 100). The EtOAc fraction was then chromatographed on Si (Silica) gel (60–120 mesh) column using CH_2_Cl_2_-MeOH (gradient) to yield 20 sub fractions. Fractions 14–18 were separately purified by Sephadex LH-20 with methanol (MeOH) to obtain compound 1 (10 mg). The fraction 19 (12.3 mg) was further chromatographed on Sephadex LH-20 and RP-18 gel column with H_2_O-MeOH (gradient) to give compound 2 (13 mg) and compound 3 (16 mg) (Figure 1).

### 4′,7-Dihydroxy-3′-methoxyflavone (Geraldone) [Compound 1]

Amorphous yellow powder, UV λmax (MeOH) (log ϵ): 236 (4.18), 316 (sh. 4.03), 337 (4.07); +AlCl3: 237 (4.12), 316 (sh. 4.06). 337 (4.13); +AlCl3 +HCl: 236 (4.15), 317 (sh. 4.08), 337 (4.14), 406 (sh. 3.16); +NaOMe: 254 (4.16), 319 (sh. 3.87), 3.68 (3.99) +NaOAc: 247 (4.19), 356 (4.17); +NaOAc +H3BO3: 236 (4.18), 317 (sh.4.04), 336 (4.17) nm; C16H12O5, M.P.: 226–228 0C; O.P.: [α]D20 -2°; 1H NMR (400 MHz, DMSO, TMS) δ ppm: 5.35-5.34 (d, 1H, J = 0.01 Hz), 5.32-5.30 (d, 1H, J = 0.02 Hz), 5.29-5.27 (d, 1H, J = 0.02 Hz), 4.68 (d, 1H, J = 0.02 Hz), 4.55 (s, 1H), 4.23-4.22 (d, 1H, J = 0.01 Hz), 3.83-3.82 (d, 1H, J = 0.01 Hz), 3.78-3.74 (m, 2H), 3.57 (s, 3H); 13C NMR (100 MHz, DMSO) δ ppm: 177.20, 150.27, 109.61, 76.74, 69.25, 60.21, 55.37, 49.89, 39.87, 38.45, 37.54, 33.87, 31.66, 30.05, 29.01, 28.70; M/Z: 455.1 (100%), 456.1 (30.23%), 281.0 (6.97%).

### 3′, 4′, 7, 8-tetrahydroxyflavanone (Isookanin) [Compound 2]

Amorphous orange powder: UV ;λmax (MeOH) (log ϵ): 239 (sh. 4.19), 280 (4.01), 378 (3.65); +NaOMe: 251 (4.01), 294 (sh. 3.78), 337 (4.16), 432 (3.47); AICI3 + HCI: 230 (sh. 4.19), 281 (4.01), 428 (3.73); AICI3: 248 (3.83), 291 (sh. 3.63), 313 (3.81), 350 (sh. 3.17), 472 (2.73); +NaOAc : 234 (sh. 4.16), 259 (4.13), 286 (4.14), 320 (3.85), 368 (3.62); +NaOAc +H_3_BO_3_: 241 (sh. 4.18), 289 (4.01), 302 (4.00), 394 (3.69) nm, M.P.: 221–224 0C; O.P.: [α]D20 -3°; 1H NMR (400 MHz, DMSO, TMS) δ ppm: 8.24(s, 1H), 4.68-4.67 (d, 1H, J = 0.01 Hz), 4.55-3.58 (d, 1H, J = 0.97 Hz), 3.53-2.99 (m, 2H), 2.98-2.01 (m, 2H), 1.99-099 (m, 3H), 093–0.62 (m, 2H); 13C NMR (100 MHz, DMSO) δ ppm: 177.18, 174.41, 150.23, 129.65, 127.70, 109.58, 76.73, 41.94, 39.25, 38.24, 33.88, 29.05, 27.12, 26.59, 17.93; M/Z: 455 (100%), 456.1 (30.23%), 284.9 (13.95%).

### 2-(3, 4-dihydroxyphenyl)-5, 7-dihydroxy-4H-chromen-4-one (Luteolin) [Compound 3]

Amorphous yellow powder; UV λmax (MeOH) (log ϵ): 252 (4.18), 268 (sh. 4.27), 341 (4.18); AICI3: 276 (4.22), 306 (sh. 3.91), 427 (4.32); +NaOMe: 231 (sh. 4.87), 261 (4.69), 327 (sh. 4.19), 401 (4.62); +AICI3 +HCI: 266 (sh. 4.12), 275 (4.18), 293 (4.10), 361 (4.13), 392 (4.13); +NaOAc 271 (4.23), 363 (4.16); +NaOAc +H_3_BO_3_: 266 (4.28), 372 (4.19) nm, M.P.: 189–194 0C; O.P.: [α]D20 -3°; 1H NMR (400 MHz, DMSO, TMS) δ ppm:4.48 (s, 1H), 3.83-3.38 (m, 2H), 2.51-2.09 (M, 2H), 1.83-1.47 (m, 2H), 1.38-1.1.09 (m, 3H); 13C NMR (100 MHz, DMSO) δ ppm: 177.18, 150.24, 129.66, 109.58, 76.73, 69.75, 41.95, 39.87, 36.68, 33.62, 31.67, 28.92, 22.09, 14.33, 13.92; M/Z: 246.8 (100%), 455 (74.41%), 285.0 (11.62%).

### Structural elucidations of the isolated compounds

Molecular formula of compound **1** was construed as C_16_H_12_O_5_ on the basis of NMR and EIMS spectral analysis. The UV spectrum exhibited characteristic absorbance bands of flavones at 236 nm and 254 nm. The compound 1 showed the M + peak at *m/z* 455.1 consistent with the presence of one hydroxyl group in ring-A and both methoxy and hydroxyl groups in ring-B, respectively. The ^1^H NMR spectrum of compound 1 depicts thirteen hydrogen atoms and showed signals due to presence of one hydroxyl group in ring-A and methoxy and hydroxyl groups are found to be present in ring-B. The ^1^H-NMR spectrum of I showed signals due to a methoxy group at δ 3.57 (3H, s), H-3 at δ 5.29-5.27 (1H, s), and two aromatic ABX coupled systems at δ 5.35 (1H, d, J = 0.01 Hz), 5.32 (1H, d, J = 0.02 Hz), and 4.68 (1H, d, J = 0.02 Hz) assigned to H-5, -6, and -8, and δ 4.23 (1H, d, J = 0.01 Hz), 3.83 (1H, d, J = 0.01 Hz), and 3.78 (1H, d, J = 0.01 Hz) assigned to H-2′, -6′, and -5′, confirming the presence of mono substitution in ring-A and disubstitution in ring-B. Based on these evidences, the compound 1 was identified as 4′, 7-Dihydroxy-3′-methoxyflavone.

The physicochemical properties of compound **2** were found to be amorphous, orange powder. The UV spectrum depicted characteristic absorbance bands of flavones at 239 and 337 nm. At position C-7 and 4, free hydroxyl groups were found which was confirmed by the presence of shift of band-II and I with NaOAc and NaOMe. The ^1^H- and ^13^C-NMR spectral data exhibited characteristic signals for the ABX system at 5H 1.99 (dd, J = 0.01 Hz, H-3 eq) and 2.98 (d, J = 0.01 Hz) and 5H 3.53 (d, J =0.02 Hz, H-2), along with an oxygen bearing methine at 6c 109.58 (C-2), a carbonyl group at 5c 127.70 (C-4) and a methylene at 5c 150.23 (C-3), assigned to positions 2 and 3 on a flavanone. The chemical shift value at 6c 109.58, corresponding to a carbonyl resonance indicated the absence of the hydroxyl group in position 5. The 1H NMR spectrum of compound **2,** two aromatic protons in the aromatic rings at δ 3.53 and 2.98 were assigned to H-5 and H-6 on ring-A. Hydroxy groups were found to be at C-7, 8, 3 and 4. Based on the above parameters compound **2** was portrayed as 3′, 4′, 7, 8-tetrahydroxyflavanone.

**Figure 2:**
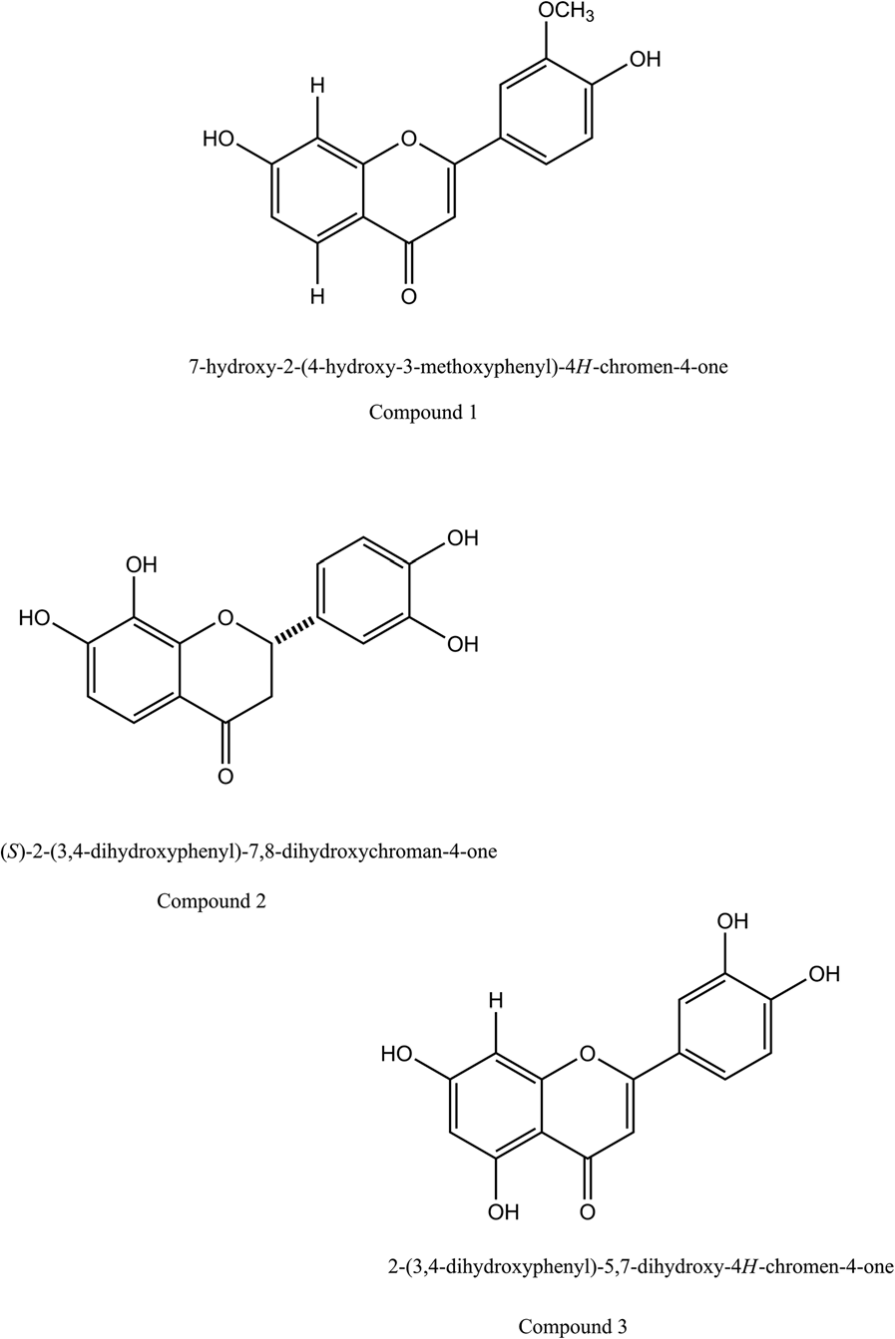
**Chemical structures of isolated flavonoids from *Albizzia Lebbeck Benth*. bark.**

The H NMR spectrum analysis of compound **3** have shown three proton signals at δ 4.48 3.83, 2.51, and 1.83 were consigned to be H2, H5 and H6 in ring-B respectively. Two meta-related (2 Hz) protons, ä 4.48 and 3.83 were assigned to H6 and H8 in ring A, respectively. In addition, a molecular ion at *m/z* 246.8 [M + H] + was detected by FAB-MS analysis. On the basis of these results, this compound **3** was recognized as 2-(3, 4-dihydroxyphenyl)-5, 7-dihydroxy-4H-chromen-4-one (Figure 2).

### In vitro intestinal α-glucosidase activity test

An inhibitory α-glucosidase activity was performed according to the method by Feng et al. [23]. In this method inhibition of α-glucosidase enzyme was determined spectrophotmetrically in a 96-well microtiter plate which is based on p-nitrophenyl-α-D-glucopyranoside (PNPG) as a substrate. Everything considered the test samples (Compound 1, 2 and 3) 120 μl each was mixed with 20 μl of enzyme solution [0.8 U/ml α-glucosidase in 0.01 M potassium phosphate buffer]. After preparing the mixture whole mixture was preincubated at 37°C prior to initiation of the reaction by incorporating the substrate. After the preincubation period of 15 min, 20 μl PNPG solutions [5.0 mM PNPG in 0.1 M potassium phosphate buffer (pH 6.8)] was incorporated and then incubated at 37°C. To terminate the reaction after 15 min of incubation, 80 μl of 0.2 M Na_2_CO_3_ in 0.1 M potassium phosphate buffer was added to the microtiter plate. The absorbance of the resultant mixture was recorded at 405 nm (Table 1).

**Table 1:** α- glucosidase and α-amylase inhibition activity of Compounds 1, 2 and 3

**Samples**	**Inhibition (%)**	
	**α-Glucosidase from saccharomyces cerevisiae**	**α-Amylase from bacillus subtilis**
Compound 1 (7-hydroxy-2-(4-hydroxy-3-methoxyphenyl)-4H-chromen-4-one)	93.91 ± 1.21***	93.98 ± 1.02***
Compound 2 ((S)-2-(3,4-dihydroxyphenyl)-7,8-dihydroxychroman-4-one)	73.14 ± 1.30**	84.36 ± 0.60***
Compound 3 (2-(3,4-dihydroxyphenyl)-5,7-dihydroxy-4H-chromen-4-one)	92.59 ± 1.36***	90.10 ± 0.58***
Acarbose	43.77 ± 1.67	37.65 ± 0.94

Statistically significant differences are designated by asterisks and double asterisks (***p < 0.001, **p < 0.01 compared to acarbose).

The results were expressed as% inhibition of α-amylase and α-glucosidase activity and calculated according to the following equation:

$$\begin{array}{c} \%\mathrm{inhibition}=100\;\times \;\\ \;(\mathrm{absorbance}\;\mathrm{of}\;\mathrm{control}–\mathrm{absorbance}\;\mathrm{of}\\ \;\mathrm{test}\;\mathrm{compounds})/\\ \;\mathrm{absorbance}\;\mathrm{of}\;\mathrm{control} \end{array}$$

### In vitro bacterial α-amylase activity test

In order to investigate the inhibitory activity of isolated compounds from *Albizzia Lebbeck Benth*. bark, α-amylase activity was performed according to the method of Chen J et al. [24]. Samples (ALD or acarbose) were mixed and pre-incubated in 20 mM sodium phosphate buffer (pH 6.7) for 5 min at 37°C. After that the volume of reaction mixture was made up to 2 ml by incorporation of 1 ml of .2% (w/v) starch dissolved in the buffer, and the whole reaction mixture was incubated for 5 min at 37°C. After the incubation, 1 ml of di-nitro salicylic acid (DNS) color reagent was incorporated and kept in boiling water bath precisely for 5 min. Then, the temperature of this mixture was decreased to room temperature by cooling it on ice and added another 6 ml of deionized water. α-Amylase activity was determined by measuring the absorbance of the mixture at 540 nm (Table 1).

### Spectrophotometric DPPH inhibition assay

Antioxidant capacities of compounds 1–3 were assessed by DPPH free radical scavenging assay. We adopted fixed reaction time assay. In the present fixed reaction time DPPH radical scavenging assay. This method is based on decline of relatively stable radical DPPH to the development of non-radical form in the presence of hydrogen donating test samples. The test samples depict antioxidant activity by the diminution in purple colored DPPH to the yellow colored diphenylpicrylhydrazine derivatives. The DPPH radical scavenging capacity was estimated according to Zhang *et al.*[25]. In this method, 2 mL of 0.1 mmol/L solution of DPPH in methanol was blended with 1 mL of each compound 1, 2 and 3. The different mixtures were incubated at room temperature in a dark room for 30 min. The absorbance was measured at 517 nm. The entire test was executed in triplicate. The scavenging capacities of different isolated compounds were calculated according to the following formula as:

$$1--\left(\mathrm{As}/\mathrm{Ac}\right)\;x\;100\%$$

Where as is the absorbance of compounds and Ac is the absorbance of control. Trolox was used as standard. The free radical scavenging capacities of different isolated compounds were expressed as μM Trolox equivalent and IC_50_ values (Table 2).

**Table 2:** DPPH radical scavenging activity of isolated Compounds 1, 2 and 3

**Samples**	**IC50 ± SD (μM)**
Compound 1 (7-hydroxy-2-(4-hydroxy-3-methoxyphenyl)-4H-chromen-4-one)	21.5*** ± 0.39
Compound 2 ((S)-2-(3,4-dihydroxyphenyl)-7,8-dihydroxychroman-4-one)	31.8** ± 0.22
Compound 3 (2-(3,4-dihydroxyphenyl)-5,7-dihydroxy-4H-chromen-4-one)	29.26** ± 0.63
Trolox	17.3 ± 1.10

Statistically significant differences are designated by asterisks and double asterisks (***p < 0.001, **p < 0.01 compared to Trolox.

### Molecular docking analysis

Mode of inhibition of Compounds 1, 2 and 3 on the α-glucosidase enzyme was further assessed by molecular docking analysis.

### Preparation of protein structure

The 3D coordinates of crystal structure of B. cereus oligo- 1,6—glucosidase (PDB ID: 1UOK) was downloaded from the RCSB protein data bank (http://www.rcsb.org/pdb) set up at Brookhaven National Laboratory in 1971. It is a worldwide repository of information about the 3D structures of large biological molecules, including proteins and nucleic acids. Water molecules were removed from the protein 1UOK before the instigation of molecular docking. The protein structure was corrected by the utilization of alternate conformations and valence monitor options as some crystallographic disorders as well as some unfilled valance atoms were present in the protein file. The resultant protein file was subjected to energy minimization by applying Chemistry at HARvard Macromolecular Mechanics (CHARMm) force fields. CHARm is a program which provides a large suite of computational tools that encompass numerous conformational and path sampling methods, free energy estimates, molecular minimization, dynamics, and analysis techniques, and model-building capabilities (http://www.charmm.org/). After the energy minimization the protein file was subjected to define and edit binding site option available on tools panel to explore the plausible binding site within the protein (1UOK). Five binding sites were explored with different fitting points. The binding site (Site 1) consist of maximum fitting point (Fitting points = 275) was selected.

### Preparation of ligand

The structures of compounds 1, 2 and 3 (4′, 7-Dihydroxy-3′-methoxyflavone, 3′, 4′, 7, 8-tetrahydroxyflavanone and Luteolin) was drawn using ChemBioDraw software. ChemBioDraw™ is a software from PerkinElmer for development of chemical structures of bioactive compounds. The prepared ligand was then subjected to add the hydrogen bonds and the energy has been minimized using CHARm force field. The ADMET properties of all the three isolated compounds which satisfy the Lipinski properties were measured primarily for the toxicity parameters using TOPKAT. Lipinski rule of five is a rule to evaluate drug likeness to determine if a chemical compound has a certain pharmacological or biological activity to make it an orally active drug in humans [26].

### Docking analysis

To find out the accurate binding model for the active site of α-glucosidase enzyme, molecular docking analysis was performed using ligand fit of GLIDE software from Schrodinger (http://www.schrodinger.com/). Molecular docking analysis was performed using crystal structure of oligo-1, 6-glucosidase (dextrin 6-alpha-glucanohydrolase, EC 3.2.1.10) from Bacillus cereus and crystal of pig pancreatic alpha-amylase. The structure of α-glucosidase (PDB: 1UOK) and crystal structure of α-amylase (PDB: 1PPI) were obtained from Protein Data Bank (http://www.rcsb.org). The mechanism of ligand position is based on the fitting points. Fitting points are incorporated into the hydrogen bonding groups on the ligand and the proteins. The ligand fit module [27] from GLIDE software was utilized to execute the molecular docking analysis, based on shape-based searching and Monte Carlo methods. At the time of docking, variable trials Monte Carlo conformation was applied where the number of steps depends on the number of rotatable bonds present in the compounds/ligands. By default the torsion number is 2, the maximum minimizations steps is 300 and maximum successive failure is 110. During the docking process the top ten conformations were engendered for each of the compound after the minimization of the energy. Each of the saved conformation was evaluated and ranked by using the scoring functions including LigScore1, LigScore2, PLP1, PLP2, Jain, PMF and Dock Score. All the compounds/ligands which forms good hydrophobic and hydrogen bond interactions with Phe163, Asp199, Val200, Phe203, Met228, Glu255, Pro257, Phe281, Asp285, Asp329 and Arg415.

## Results and discussion

Among all of the plant constituents, flavonoids are the most scrutinized group for their effect on the health concerns. A recent study supports the efficacy of flavonoids in the treatment of diabetes mellitus by influencing the β-cell mass and function as well as insulin signaling [28]. Several researches suggested having beneficial and shielding effect against cancer [29] stress, hypertension and heart disorders [30,31].

The flavonoids are low molecular weight bioactive phenols actively participate in the cell wall synthesis [32-35]. Flavonoids were revealed to regulate insulin secretion, insulin signaling, carbohydrate digestion and glucose uptake in insulin-sensitive tissues through various intracellular signaling pathways [36]. One recent study has assessed the relationship between the flavonoids and the risk of diabetes. This study confirms that the men and women received the flavonoids were at lower risk of diabetes [37].

Intestinal α-glucosidases (maltase, isomaltase and sucrase) are membrane bound enzymes integrated in the epithelium of the small intestine and are implicated for the final step of carbohydrate hydrolysis to produce absorbable monosaccharide. Starch is the major dietary carbohydrate source of glucose and diabetic complications depends upon the rate and extent of digestion of glucose from starch by the pancreatic α-amylase and intestinal α-glucosidase [38]. Disaccharides and oligosaccharides are broken down by the pancreatic α-amylase before the intestinal α-glucosidase enzyme catalyzes the breakdown of disaccharides into glucose which is then later absorbed into the systemic circulation. Inhibition of these enzymes would slow down the breakdown of starch into the simpler saccharides in the gastrointestinal tract, thus reducing the postprandial hyperglycemia [39,40]. α-Glucosidase inhibitors were also reveled to possess property to prevent dysfunction of β-cell insulin secretion in diabetic patients [41,42].

In the present research exertion α-glucosidase inhibitory activity was determined against the enzyme obtained from yeast (S. cerevisiae). α-amylase was procured from bacteria (*B. subtilis*). Acarbose (10 mg/ml) was used as standard. The results clearly suggest that flavonoids obtained from *Albizzia Lebbeck Benth.* bark i.e. compounds 1–3 inhibited the α-glucosidase and α-amylase to a significant extent (Table 1).

**Figure 3:**
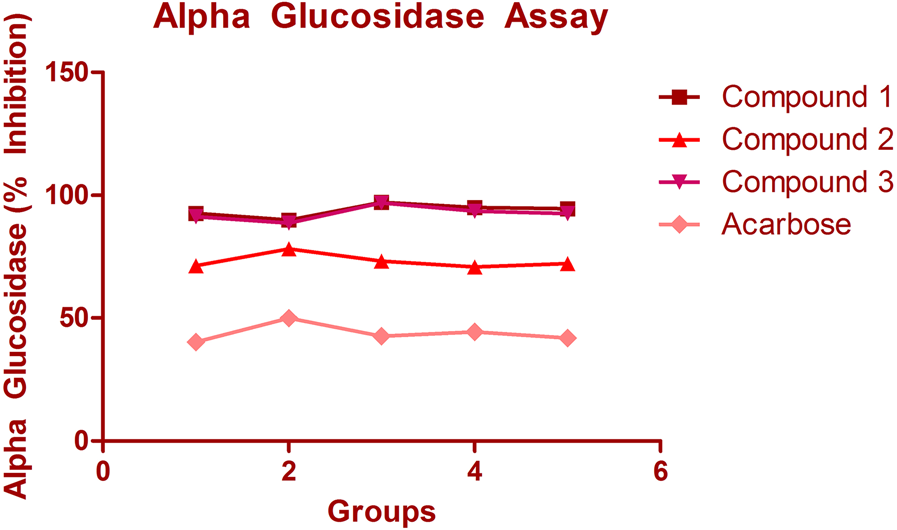
Alpha glucosidase assay for compounds 1, 2 and 3.

**Figure 4:**
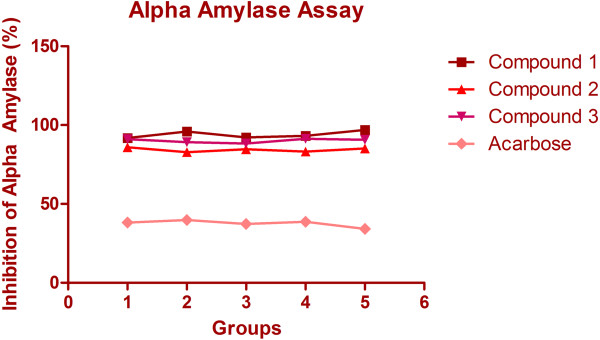
Alpha amylase assay for compounds 1, 2 and 3.

In the α-glucosidase inhibition assay, all the isolated compounds viz. compound 1(7-hydroxy-2-(4-hydroxy-3-methoxyphenyl)-4H-chromen-4-one), compound 2((S)-2-(3,4-dihydroxyphenyl)-7,8-dihydroxychroman-4-one) and compound 3 (Compound 3: (2-(3,4-dihydroxyphenyl)-5,7-dihydroxy-4H-chromen-4-one) strongly inhibited the α-glucosidase as compared acarbose (p < 0.001) (Figure 3). Furthermore, compound 1 exhibits the strongest inhibitory activity when weighed against the other two isolated compounds and acarbose. In the α-amylase inhibition assay, the compounds 1 showed higher inhibitory activity (93.98%) as compared to compound 2, 3 and acarbose (p < 0.001) (Table 1, Figure 4). Our results evidently demonstrate that isolated compounds 1–3 inhibited the α-glucosidase and α-amylase to an enormous extent.

**Figure 5:**
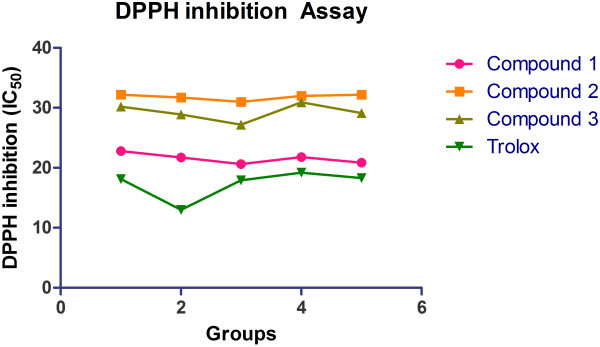
DPPH inhibition assay for compounds 1, 2 and 3.

**Figure 6:**
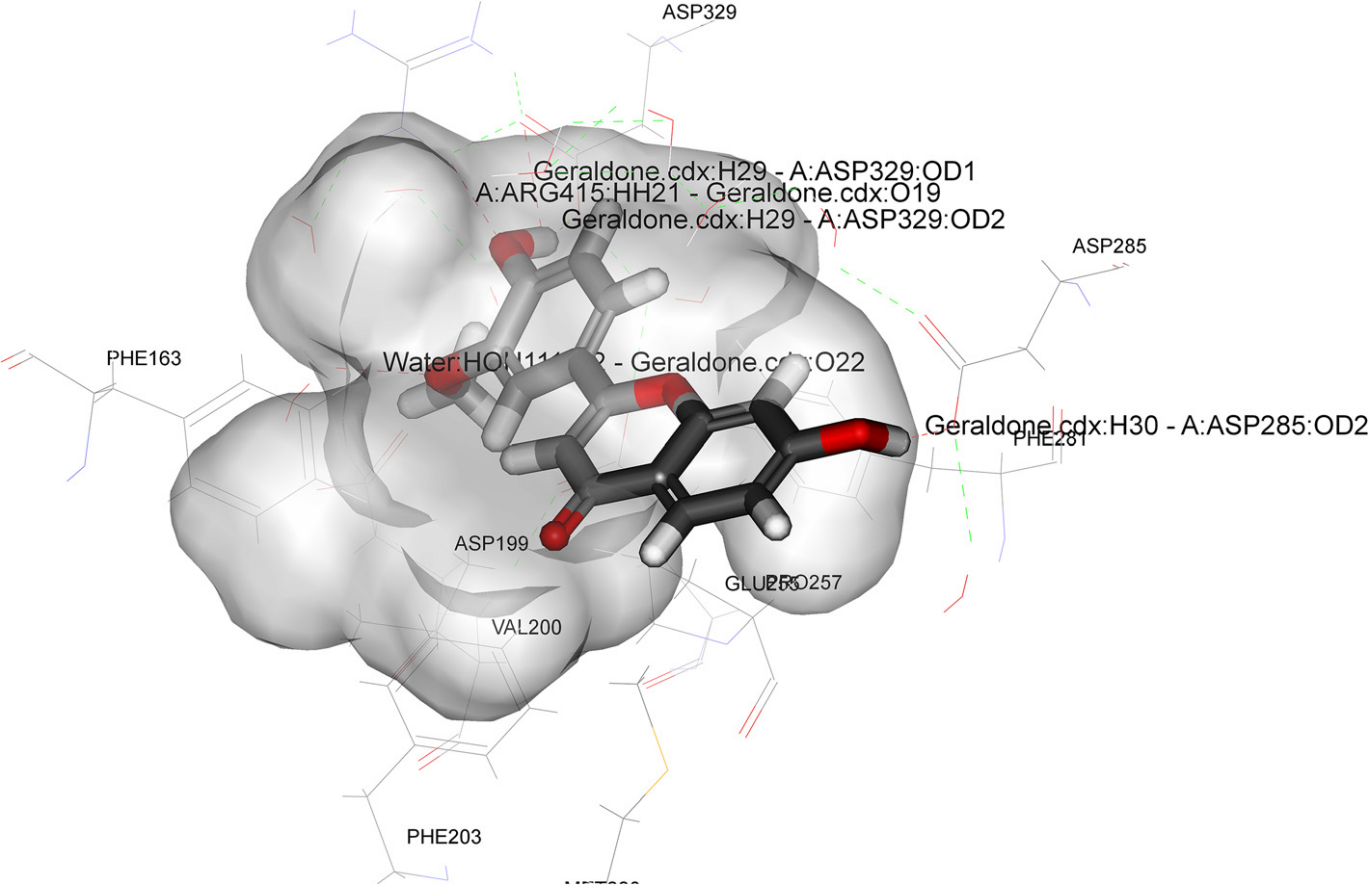
**Close view of binding mode of validated active ligand (Compound 1) in α-glucosidase enzyme binding pocket.** Hydrogen bonds are represented by green dotted lines.

**Figure 7:**
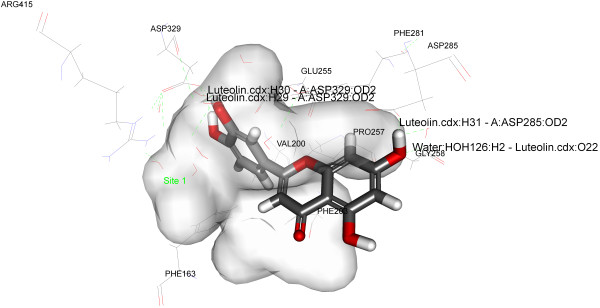
**Close view of binding mode of validated active ligand (Compound 2) in α-glucosidase enzyme binding pocket.** Hydrogen bonds are represented by green dotted lines.

**Figure 8:**
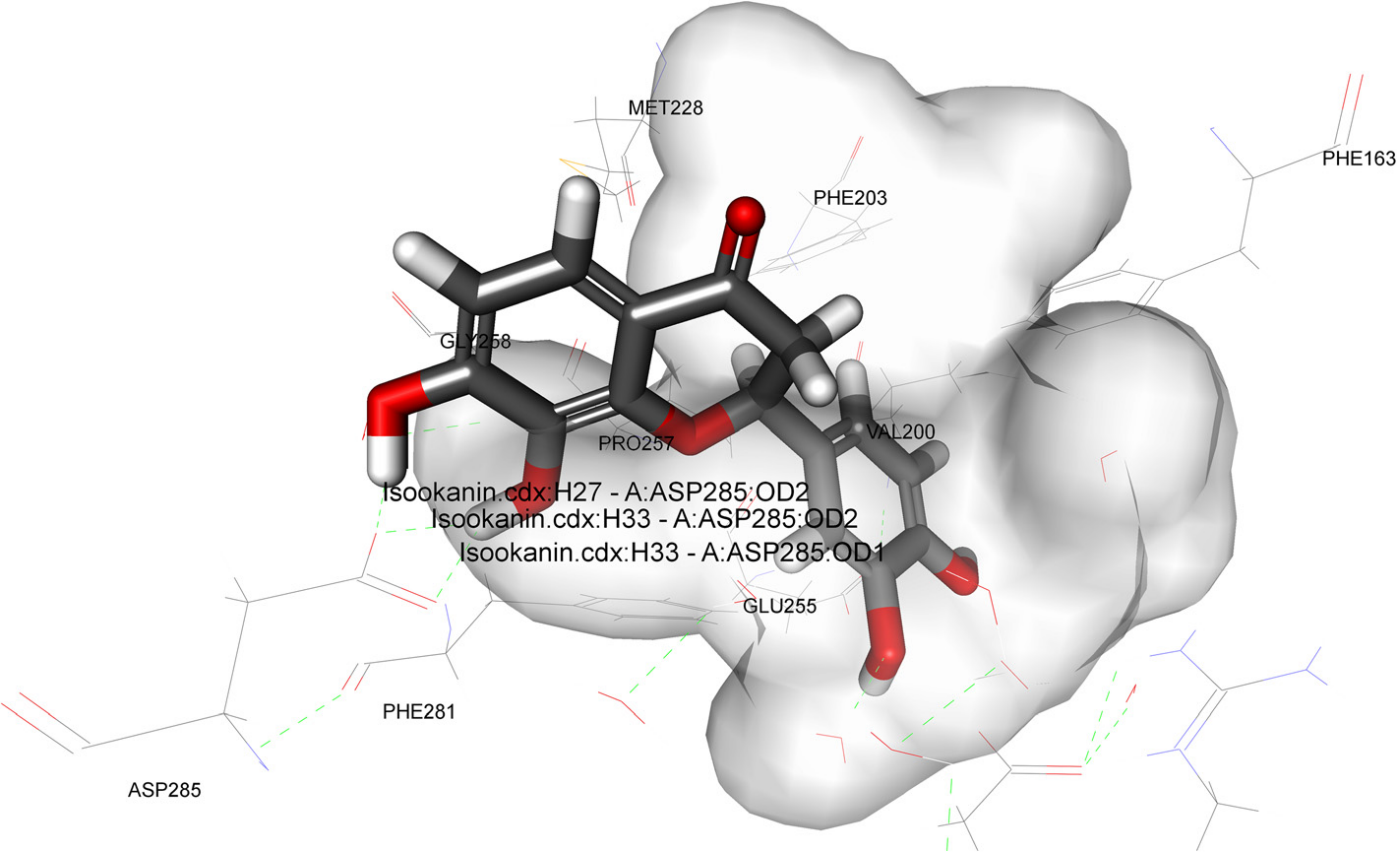
**Close view of binding mode of validated active ligand (Compound 3) in α-glucosidase enzyme binding pocket.** Hydrogen bonds are represented by green dotted lines.

**Figure 9:**
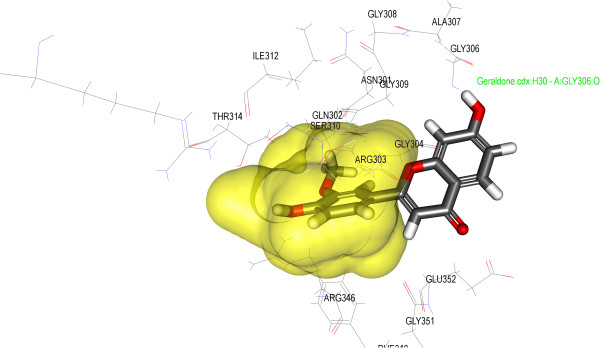
**Close view of binding mode of validated active ligand (Compound 1) in α-amylase enzyme binding pocket.** Hydrogen bonds are represented by green text.

Antioxidants have been known to exert the protective effects against the oxidative damage and are associated with reduced risk of chronic diseases [43]. Both endogenetic and exogenously formed free radicals can lead to carcinogenesis, aging, inflammation, diabetes and atherosclerosis when react with the cellular biological molecules [44]. A research has shown a postprandial increase in the concentration of free radicals in the type-II diabetic patients [45]. Capacity to scavenge the DPPH-radicals is the basis of a common antioxidant assay. There are researches that prove that antioxidants can protect against the damage caused by free radicals that have been a concern in the etiology of a large number of diseases [46]. Therefore, we have taken the present research endeavor to evaluate the free radical scavenging capacities of isolated flavonoids. The free radicals, DPPH, scavenging capacities of different isolated compounds were evaluated and all the compounds were found to scavenge DPPH radical to a certain extent. However compound 1 showed highest activity at IC_50_ 21.5 μM among the other two isolated compounds when compared to positive control Trolox (Table 2, Figure 5). The result of the docking simulation of compounds 1–3 and α-glucosidase and α-amylase are shown in Figures 6, 7, 8 and 9. Binding pocket of α-glucosidase enzyme was found to be composed of PHE163, ASP199, VAL200, PHE203, MET228, GLU255, PRO257, PHE281, ASP285, ASP329 and ARG415. Hydrogen bonds and hydrophobic-contacts were again the most important type of interactions with the enzyme.

Figure 6 displayed the interaction of compound 1 with yeast α-glucosidase enzyme. Polar amino acid residues i.e. ASP329 and ARG415 have strong H-bonding with the acetate group of the ligand. Compound 1 showed the strongest binding affinity with protein as incidental by its lowest internal energy (-3.53 kcal/mol), values are given in the Table 3. This observation is in good agreement with the observation with the α-glucosidase assay where compound 1 exhibits the highest inhibition (%) for the particular enzyme. In the predicted binding mode the hydroxy group of the compound 2 interacts with the ASP285 directly by a hydrogen bond at H27 and H33. The water molecules were omitted in the docking simulation in order to explore the additional docking pose without excluding the possibility of direct hydrogen bonding. Compound 3 also binds to the polar amino acid residues ASP329 and ASP285 with strong hydrogen bonding. Molecular docking of compounds 1–3 with α-glucosidase and α-amylase predicted that these compounds adopt a similar conformation and bind in similar site as acarbose. Molecular docking simulations of compounds 1–3 was performed with α-amylase (PDB: 1PPI). Binding pockets of α-amylase was explored to be compiled of ARG267, ASN301, GLN302, ARG303, GLY304, GLY306, ALA307, GLY308, GLY309, SER310, ILE312, THR314, ARG346, PHE348, GLY351, GLU352. During the molecular docking of compound 1 with α-amylase, hydrogen bonds were observed between the methoxy group of compound 1 and polar amino acid residue GLY306 (residue involved in the catalytic domain). The affinity of compounds 2 and 3 was weakest which can be attributed to the lesser number of rotatable bonds present in the compounds 2 and 3 (Table 4, Figures 7, 8 and 9). Furthermore, the *in silico* results supported the outcome from experimental enzymatic assays.

**Table 3:** Docking results based on the LigScore1, LigScore2, PLP1, PLP2, Jain, PMF and Dock Score of the compounds 1, 2 and 3 into the α-glucosidase enzyme (PDB: 1UOK)

**S. no.**	**Compound**	**LigScore1**	**LigScore2**	**PLP1**	**PLP2**	**JAIN**	**PMF**	**Dock score**	**Internal energy (kcal/mol)**
1	4′,7-Dihydroxy-3′-methoxyflavone (Compound 1)	4.03	3.43	48.65	49.25	2.1	125.25	61.18	-3.53
2	3′,4′,7,8-tetrahydroxyflavanone (Compound 2)	4.08	4.19	43.78	49.02	1.03	86.89	68.86	-3.189
3	Luteolin (Compound 3)	4.7	4.47	43.67	47.4	-0.15	94.85	66.91	-2.423

**Table 4:** Docking results based on the LigScore1, LigScore2, PLP1, PLP2, Jain, PMF and Dock Score of the Compounds 1, 2 and 3 into the α-amylase enzyme (PDB: 1PPI)

**S. no.**	**Compound**	**LigScore1**	**LigScore2**	**PLP1**	**PLP2**	**JAIN**	**PMF**	**Dock score**	**Internal energy (kcal/mol)**
1	4′,7-Dihydroxy-3′-methoxyflavone (Compound 1)	-999.9	-999.9	58.52	49.39	0.97	23.52	31.63	1.452

As concluding remarks, the isolated flavonoids from *Albizzia Lebbeck Benth.* bark showed a high potential as inhibitors of α-glucosidase, α-amylase and DPPH. In-vitro evaluation of these compounds clearly depicts the inhibition of both the enzymes responsible for high blood glucose level as well as the inhibition of formation of ROS (Reactive Oxygen Species) accountable for the development of type-II diabetes. Inhibition of these enzymes was further supported by the molecular docking analysis. Thus 7-hydroxy-2-(4-hydroxy-3-methoxyphenyl)-4H-chromen-4-one, (S)-2-(3,4-dihydroxyphenyl)-7,8 dihydroxychroman-4-one) and (2-(3,4-dihydroxyphenyl)-5,7-dihydroxy-4H-chromen-4-one) showed to the potential to decrease the fasting and postprandial glucose levels in type-II diabetes patients.

## Conclusion

Our *invitro* experimentation and molecular docking studies results demonstrated that isolated flavonoids from *Albizzia Lebbeck Benth*. bark significantly inhibits the α-glucosidase, α-amylase and DPPH enzymes. Therefore, the present study suggests a basis for the possible use of *Albizzia Lebbeck Benth*. bark as functional food. Consequently, *in-vivo* antidiabetic research exertion of isolated flavonoids is underway in our laboratory to explore the other plausible mechanism of action to restrain the hyperglycemia and hyperlipidemia in the STZ (streptozotocin) induced diabetic rats.

## Competing interest

The authors declare that they have no competing interests.

## Authors’ contributions

DA envisages and carried out the research exertion. VK and MS helped in execution of the experiments. AV partakes in the design of the experiments and interpretation of the spectral data of the compounds. All authors read and approved the final manuscript.

## Pre-publication history

The pre-publication history for this paper can be accessed here:

http://www.biomedcentral.com/1472-6882/14/155/prepub
